# Triple negative breast cancer: approved treatment options and their mechanisms of action

**DOI:** 10.1007/s00432-022-04189-6

**Published:** 2022-08-17

**Authors:** Aditya Mandapati, Kiven Erique Lukong

**Affiliations:** Biochemistry, Microbiology and Immunology, College of Medicine, University of Saskatchewan, 107 Wiggins Road, Saskatoon, SK S7N 5E5 Canada

**Keywords:** Breast cancer, Taxol, Doxorubicin, Epirubicin chemotherapy, PARP inhibitors, Olabarib, Atezolizumab, Immunotherapy, Keytruda, BRCA1 and BRCA2, ER, HER2, TNBC

## Abstract

**Purpose:**

Breast cancer, the most prevalent cancer worldwide, consists of 4 main subtypes, namely, Luminal A, Luminal B, HER2-positive, and Triple-negative breast cancer (TNBC). Triple-negative breast tumors, which do not express estrogen, progesterone, and HER2 receptors, account for approximately 15-20% of breast cancer cases. The lack of traditional receptor targets contributes to the heterogenous, aggressive, and refractory nature of these tumors, resulting in limited therapeutic strategies.

**Methods:**

Chemotherapeutics such as taxanes and anthracyclines have been the traditional go to treatment regimens for TNBC patients. Paclitaxel, docetaxel, doxorubicin, and epirubicin have been longstanding, Food and Drug Administration (FDA)-approved therapies against TNBC. Additionally, the FDA approved PARP inhibitors such as olaparib and atezolizumab to be used in combination with chemotherapies, primarily to improve their efficiency and reduce adverse patient outcomes. The immunotherapeutic Keytruda was the latest addition to the FDA-approved list of drugs used to treat TNBC.

**Results:**

The following review aims to elucidate current FDA-approved therapeutics and their mechanisms of action, shedding a light on the various strategies currently used to circumvent the treatment-resistant nature of TNBC cases.

**Conclusion:**

The recent approval and use of therapies such as Trodelvy, olaparib and Keytruda has its roots in the development of an understanding of signaling pathways that drive tumour growth. In the future, the emergence of novel drug delivery methods may help increase the efficiency of these therapies whiel also reducing adverse side effects.

## Introduction

Breast cancer (BC) is the most frequently diagnosed cancer worldwide with an estimated 2.3 million new cases in 2020, representing 11.7% of all cancer cases. BC accounted for 684,996 deaths in 2020 (Sung et al. 2020). In Canada, for example, 27,700 cases were diagnosed in 2021 with an estimated 5400 deaths as a result (Canadian Cancer Statistics 2021). In the United States of America (USA) in 2021, a total of 290,560 new cases of BC (287,850 females and 2710 males) and 44,130 deaths (43,250 females and 530 males) were reported (Siegel et al. 2022). While breast cancer is the most diagnosed type of cancer in females worldwide, in developed countries such as the USA, Canada, Australia, and England, lung cancer causes more deaths (Bray et al. 2018; Lukong 2017). Since the 2000s, the reduction in mortality due to breast cancer can be attributed to an increase in screening, awareness, and development of effective therapies made available in these regions (Bray, et al. 2018; Lukong 2017). Breast cancer develops from the abnormal proliferation of mammary epithelial cells to form carcinoma in situ which can then become invasive and metastatic (Makki 2015; Place et al. 2011). These tumors can be classified using either histological or molecular features.

Molecular subtype classification involves the assessment of the presence or absence of ER, PR, and HER2 (Perou et al. 2000; Sørlie et al. 2001). These subtypes are luminal A, luminal B, HER2-positive, and Triple-negative breast cancers (TNBCs) (Lukong 2017; Fragomeni et al. 2018). Luminal A breast cancers constitute ~ 30–40% of all invasive cases (Fragomeni et al. 2018). These tumors are ER-positive, PR-positive but HER2-negative and are generally low grade (Fragomeni et al. 2018). Luminal B tumors consist of 20–30% of all cases and are typically ER-positive, PR-negative/positive, and HER2-negative/positive (Fragomeni et al. 2018). They are also higher grade, with a poorer prognosis than luminal A cases (Fragomeni et al. 2018). HER2-positive breast cancers have two subcategories: HER2-enriched (ER and PR-negative but HER2-positive) and luminal HER2 (ER and PR-positive and HER2-positive) (Kneubil et al. 2013; Vici et al. 2015). Overall, 12–20% of breast cancer cases are classified as HER2-positive (Fragomeni et al. 2018). Triple negative breast cancer (TNBC) is diagnosed in 15–20% of all breast cancer patients and is defined by the absence of ER, PR, and HER2 expression (Fragomeni et al. 2018; Foulkes et al. 2010).

Patients with TNBCs are more predisposed to adverse outcomes, recurrence, and metastasis than patients with other breast cancer subtypes. Patients with TNBC were significantly more likely to die within 10 years of diagnosis than patients with other breast cancer subtypes (Dent et al. 2007). In an analysis of 1025 breast cancer-specific deaths, patients with the TNBC subtype were found to have significantly reduced survival probability compared to patients with hormone receptor-positive or negative tumors (Lin et al. 2012). This difference was observed even after adjustment for risk factors such as age, race, and tumor size (Lin et al. 2012). Dent and colleagues found that 33.9% of TNBC patients experienced recurrence and metastasis compared to 20.4% of patients with other breast tumors (Dent et al. 2007). They reported that these metastases were more likely to be locoregional at first before spreading to distant tissue (Dent et al. 2007). Furthermore, TNBC metastases were more likely to occur in visceral organs and soft tissue, as reported by Liedtke et al. (Liedtke et al. 2008). An analysis by Lin and scientists found that first recurrences were most likely to be located in the brain, lung, or locoregional sites (Lin et al. 2012). Of these sites, the central nervous system appeared to be the most prevalent, with first and subsequent recurrences of TNBC occurring in the TNBC in 174 out of 480 TNBC patients analyzed (Lin et al. 2012). These studies also reported that TNBC tumors were less likely to recur in the bone than hormone receptor-positive breast tumors (Dent et al. 2007; Lin et al. 2012; Liedtke et al. 2008).

The development of TNBCs can be attributed to several risk factors including genetic predisposition, race, age, obesity, BRCA1 and BRCA2 mutations are considered risk factors for the development of TNBC tumors (Schneider et al. 2008). A meta-analysis of 46.870 patients, including 868 carriers of BRCA1 mutations found that patients carrying the tumors were significantly more likely to develop TNBC than both non-carriers as well as patients carrying BRCA2 mutations (Chen et al. 2018). Additionally, African American women are more likely to develop TNBC and have adverse outcomes than European–Americans (Lin et al. 2012; Prakash et al. 2020). Out of 190 TNBC patients, it was found that a significant proportion were either African American or Asian women compared to patients without TNBC (Yeh et al. 2017). The analysis also found that a prior history of breast cancer was a strong risk factor associated with TNBC development in these patients (Yeh et al. 2017). The subtype of breast cancer also develops more frequently in women under the age of 50 and is associated with obesity though studies on the latter risk factor have shown contrasting results (Lin et al. 2012; Dolle et al. 2009). While there was a higher prevalence of TNBC in obese premenopausal women compared to their normal-weight counterparts, Body Mass Index (BMI) had no apparent effect on the risk of TNBC development in postmenopausal women (Lin et al. 2012).

The treatment of breast cancer is dictated by the molecular subtype of the tumor. Luminal A and B carcinoma overexpress ER and are therefore treated by targeting the estrogen receptor pathway using endocrine therapy (Hanker et al. 2020). Endocrine therapies include Selective Estrogen Receptor Modulators (SERMs) such as Tamoxifen, Selective Estrogen Receptor Downregulators (SERDs) such as Fulvestrant, and Aromatase Inhibitors (AIs) like Letrozole (Hanker et al. 2020). Generally administered after surgery, these drugs have drastically reduced mortality and remission rates in patients since their introduction (Hanker et al. 2020). HER2-positive cases are treated with HER2-targeting agents trastuzumab and lapatinib (Arteaga et al. 2011). To improve the efficiency of both endocrine and HER2-targeting therapies, combinatorial approaches using either of these agents along with PI3K inhibitors, CDK 4/6 inhibitors, or PD1 inhibitors are being tested (Hanker et al. 2020; Arteaga et al. 2011).

On the other hand, TNBC tumors present a significant challenge to the development and use of targeted therapies due to the lack of ER, PR, and HER2 expression (Pareja et al. 2016). The term TNBC was first used to describe cases that only responded to chemotherapy (Brenton et al. 2005). Despite the absence of these biomarkers, TNBCs are heterogenous, with various subtypes, each distinguished by the up or downregulation of different protein pathways (Bianchini et al. 2016). TNBC cases are classified into basal-like 1 (BL1), basal-like 2 (BL2), immunomodulatory (IM), mesenchymal (M), mesenchymal stem-like (MSL), and luminal androgen receptor (LAR) subtypes (Pareja et al. 2016; Lehmann et al. 2016).

Regardless of the subtype, TNBC patients have primarily been treated with cytotoxic chemotherapy either before (neoadjuvant) or after surgery (adjuvant) (Bianchini et al. 2016). TNBC cases often benefit from chemotherapy to a greater degree than other breast cancer subtypes (Carey et al. 2007). For example, a study by von Minckwitz and colleagues showed that neoadjuvant chemotherapy using anthracyclines and taxanes benefited 30–40% of patients with early-stage TNBC (Minckwitz et al. 2012). Apart from anthracyclines such as doxorubicin (Adriamycin) and taxanes such as paclitaxel (Taxol), the FDA has approved PARP inhibitors such as olaparib (Lynparza) and immunotherapy such as atezolizumab (Tecentriq) to be used in combination with the chemotherapy agents (Pareja et al. 2016; Collignon et al. 2016; Schmid et al. 2018). More recently, the immune-targeted therapy Keytruda was fast-tracked for approval to treat metastatic triple negative breast cancer (Raedler and Keytruda (Pembrolizumab) 2015).

Due to the lack of biomarkers for targeted therapies and an aggressive, often invasive, disease progression, there are few FDA-approved drug treatments for triple-negative breast cancer. This review describes the mechanisms of action of these therapies as well as the limited scope of treatments available for TNBC to open avenues for future drug development.

## TNBC subtypes

*BRCA* (BReast CAncer) genes (*BRCA 1* and *2* or *1/2)* are tumor-suppressor genes that play a role in DNA damage repair and mutations of these genes that results in defective DNA repair mechanisms may increase the risk of developing breast cancer (Venkitaraman 2014). TNBCs with germline BRCA1/2 can be referred to as “BRCAness” tumors (Summa et al. 2013). The prevalence of *BRCA1/2* among women with TNBC varies significantly by ethnicity/race and age (Hahnen et al. 2017; Lukong et al. 2017). This means that not all TNBCs are equal.

Gene expression studies have revealed that TNBC is a heterogeneous subgroup of tumors that is also distinguishable by the up or downregulation of different cellular pathways (Bianchini et al. 2016). A landmark study by Lehmann and colleagues led to the classification of TNBC into seven distinct subtypes based on gene expression profiling (Lehmann et al. 2016). These include six stable subtypes: (i) basal-like 1 (BL1), (ii) basal-like 2 (BL2), (iii) immunomodulatory (IM), (iv) mesenchymal (M), (v) mesenchymal stem-like (MSL) and (vi) luminal androgen receptor (LAR) subtypes, and an unstable (UNS) subtype (vii), together referred to sometimes as Lehmann TNBC subtypes (Pareja et al. 2016; Lehmann et al. 2016). BL1 breast cancers overexpress genes in the DNA damage response pathway and are stained by Ki67 while BL2 tumors are associated with the upregulation of growth factor signaling, TP53, and myoepithelial markers (Fragomeni et al. 2018; Pareja et al. 2016). IM tumors are defined by hyperactive immune signaling cascades and infiltration of lymphocytes into the tumor (Fragomeni et al. 2018; Pareja et al. 2016). Mesenchymal and mesenchymal stem-like TNBCs share several similarities (enrichment of epithelial-to-mesenchymal transition genes) but are differentiated by the increase in expression of mesenchymal stem cell genes in the latter subtype (Pareja et al. 2016). The LAR subtype, as the name suggests, presents similarly to luminal breast cancers due to androgen receptor activation (Pareja et al. 2016). It is important to note that Lehmann et al. later revised their previous sub-classification to include only four subtypes (TNBCtype-4): BL1, BL2, M, and LAR because the immunomodulatory signature for instance occurred in all the other TNBC molecular subtypes, making up about 20% of all TNBCs (Lehmann et al. 2016). Studies by Burstein et al. also led to the classification of four TNBC subtypes that comprised LAR, mesenchymal (MES), basal-like immunosuppressed (BLIS) and basal-like immune-activated (BLIA) (Burstein et al. 2015). No surprisingly, BLIA showed a better prognosis compared with BLIS based on its more favourable immunological background (Burstein et al. 2015).

### Targeting TNBC subtypes

Classification of TNBC subtypes can help guide therapy selection. BL1 and BL2 tumors, for example, are particularly susceptible to PARP inhibitors due to the dysregulation of DNA damage repair that characterizes these cancers (Yin et al. 2020). Additionally, the increased activity of growth factor receptors, which differentiates BL2 from BL1 tumors, makes anti-growth factor drugs such as Lapatinib, Gefitinib, and Cetuximab ideal candidates to treat BL2 TNBCs (Yin et al. 2020). Both BL1 and BL2 cancers respond to platinum-based chemotherapy (Maqbool et al. 2022). The M and MSL subtypes can be targeted using the mTOR inhibitor Rapamycin with M-type TNBCs being susceptible to growth factor inhibition and the MSL-type being susceptible to the Src inhibitor dasatinib (Maqbool et al. 2022). Immunomodulatory TNBCs highly express immune system-associated genes which makes them vulnerable to anti-immune checkpoint therapeutics such as PD1 and PDL1 inhibitors (Atezolizumab and Pembrolizumab, for example) (Yin et al. 2020). Finally, LAR subtype TNBCs can be targeted using anti-androgen receptor therapies such as Bicalutamide and Enzalutamide (Barton et al. 2016). A summary of the various molecular subtypes of TNBC and their corresponding therapies is shown in Table 1.

**Table 1:** Summary of TNBC subtypes, their characteristics and treatments that may be used to treat them

TNBC subtype	Molecular characteristics	Possible treatment options
Basal-Like 1 (BL1)	BRCA1/BRCA2 mutations, TP53 mutations, Cell cycle gene expression (RB1, CDKN2A), DNA repair pathway dysfunction	PARP inhibitors (Olaparib, Talazoparib), Platinum-based chemotherapy (Cisplatin)
Basal-Like 2 (BL2)	Activation of EGFR, MET, IGF-1R and Wnt/β-catenin signaling, Expression of glycolysis and gluconeogenesis genes, Myoepithelial factor expression (TP63 and MME)	PARP Inhibitors (Olaparib, Talazoparib), Growth factor inhibitors (Lapatinib, Gefitinib and Cetuximab), Platinum-based chemotherapy (Cisplatin)
Mesenchymal (M)	Activation of cell migration pathways, Increased extracellular matrix (ECM) interaction, Differentiation pathway dysregulation (Wnt signaling, TGF-β signaling)	mTor inhibitors (Rapamycin), Growth factor inhibitors (Lapatinib, Gefitinib and Cetuximab),
Mesenchymal Stem-Like (MSL)	Reduced cell proliferation and cell cycle gene expression, Increased expression of stemness genes (HOX, NGF receptor, VCAM1)	Abl/Src inhibitor (Dasatinib), mTOR inhibitor (Rapamycin),
Immunomodulatory (IM)	Activation of genes associated with immune system cells (Th1/Th2 pathway, IL2 and IL7 pathways, NK cell pathway etc.), Increased antigen presentation	Immune checkpoint inhibitors that target PDL1 or PD1 (Atezolizumab and Pembrolizumab) PARP inhibitors (Olaparib, Talazoparib) Platinum-based chemotherapy (Cisplatin)
Luminal Androgen Receptor (LAR)	High expression of androgen receptor (AR), Increased hormone receptor signaling (including androgen/estrogen metabolism)	Anti-AR therapy (Bicalutamide, Enzalutamide)

Adapted from Yin et al. (2020), Maqbool et al. (2022) and Barton et al. (2016)

## FDA-approved TNBC therapies

### PARP inhibitors

In their landmark study in 2005, farmer and colleagues showed that embryonic stem cells deficient in *BRCA1* and/or *BRCA2* were particularly susceptible to cell death when subjected to small-molecule PARP inhibitors (Farmer et al. 2005). Due to the prevalence of deleterious, germline *BRCA* mutations in TNBC, the study opened possibilities to target PARP and treat these cancers specifically, leading to the development of Lynparza and Talzenna (Collignon et al. 2016; Farmer et al. 2005). In 2018, the FDA approved Lynparza (manufactured by AstraZeneca) and Talzenna (manufactured by Pfizer) for the treatment of germline BRCA-mutated, metastatic triple negative breast cancer (U.S. 2018a).

Initially developed and approved for the treatment of germline *BRCA*-mutated ovarian cancer in 2014, Lynparza (generic name olaparib) was then tested on patients with HER2-negative metastatic breast cancer in a randomized, open-label, phase 3 clinical trial (named OlympiAD) in 2017 (Kim et al. 2022; Robson et al. 2017). Patients who received olaparib showed significantly longer progression-free survival versus patients who were given traditional chemotherapy (Robson et al. 2017). The trial indicated that olaparib monotherapy reduced the risk of death or disease progression by 42% (Robson et al. 2017). Similarly, Talzenna (generic name talazoparib) was tested on patients with advanced, HER2-negative breast cancer in the EMBRACA phase 3 clinical trial in 2017 (Litton et al. 2018). The trial showed that talazoparib treatment increased progression-free survival by 3 months compared to traditional chemotherapy in these patients (Litton et al. 2018). Lynparza and Talzenna have different chemical structures but share the same mechanism of action in that they target and inhibit the role of PARP in the DNA damage response pathway (Shen et al. 2015).

#### Poly (ADP-ribose) polymerases (PARPs)

Poly (ADP-ribose) polymerases (PARPs) are a family of related enzymes that covalently add poly (ADP-ribose) chains onto their targets, in a process called PARylation (Schreiber et al. 2006). PARPs play key roles in various cellular processes that include transcription, replication, recombination, and notably, DNA repair. Among PARP family members, PARP1 is the primary DNA damage sensor and generates about 90% of poly (ADP-ribose) chains following DNA damage. PARP1 contains six functional domains, which include three zinc finger-related domains (DNA binding domains), one BRCA1 C-terminus domain (auto-modification domain), a tryptophan-/glycine-/arginine-rich domain (WGR domain), and one catalytic domain (Krishnakumar and Kraus 2010). Further, the catalytic domain of PARP1 is made up of two subdomains: a helical domain (HD) and an ADP-ribosyltransferase catalytic domain (ART) (Krishnakumar and Kraus 2010). The (ADP-ribose) polymerase activity of PARP1 is strongly regulated by interaction with single-stranded DNA breaks. PARP1 recognizes and interacts with DNA single-strand breaks via its zinc finger-related domains. In its inactive, non-DNA binding status, the HD of PARP1 inhibits the binding between PARP1 and its cofactor β-nicotinamide adenine dinucleotide (β-NAD) via the ART subdomain. PARP1 binds to SSBs, thus abrogating the auto-inhibitory function of HD and resulting in the activation of ART (Rouleau et al. 2010). Once on the SSBs, PARP1 recruits scaffolding proteins such as DNA ligase III and DNA polymerase β (Lee et al. 2014). PARP1 also interacts with PARG (poly ADP-ribose glycohydrolase) to attach ADP-ribose moieties to histones, facilitate chromatin remodeling, and recruit DNA damage repair proteins (Livraghi and Garber 2015). Once the chromatin is unwound, the assembled repair complex uses the undamaged template strand to fix the break (Livraghi and Garber 2015). Auto-PARylation of PARP1 releases PARP1 from the repaired DNA, thus reinstituting the auto-inhibitory status (Rouleau et al. 2010). In the presence of PARP inhibitors, the PARP-dependent DNA repair system cannot be activated leading to the development of double-strand breaks and susceptibility of BRCA1/2-mutant breast cancer cells for instance to synthetic lethality (Farmer et al. 2005; Bryant et al. 2005). Additionally, studies have shown that PARP1 recognition of DNA damage also facilitates the homologous recombination repair (HR) pathway, though the enzyme does not play an active role in HR beyond binding to the double-stranded break site (Hay et al. 2009; Schultz et al. 2003). In the presence of PARP inhibitors (PARPi), the PARP-dependent DNA repair system cannot be activated. Disruption of both HR and the PARP-mediated base excision repair (BER) pathways through PARP inhibition is often lethal to cells (Farmer et al. 2005). BRCA1/2-mutant breast cancer cells for instance are susceptible to PARPi-induced synthetic lethality (Farmer et al. 2005; Bryant et al. 2005).

#### Mechanism of action of PARP inhibitors: lynparza and talzenna

The mechanism of action of PARP inhibitors in tumors is inextricably linked with *BRCA1/2* (Farmer et al. 2005; Bryant et al. 2005). PARP-deficient mice do not develop tumors and are otherwise healthy and fertile but the inhibition of PARP in *BRCA1/2* deficient cancer cells has been shown to cause cell death (Farmer et al. 2005; Bryant et al. 2005; Conde et al. 2001). Therefore, it is important to understand the function of *BRCA1* and *BRCA2* (in DNA damage response) to appreciate why germline *BRCA* mutated breast cancers are susceptible to PARP inhibition (D'Andrea 2018).

The BRCA proteins play a role in repairing double-stranded DNA breaks through the process of homologous recombination repair (HR) (Byrum et al. 2019). BRCA1 and BRCA2 migrate to genomic lesions and interact with proteins such as BARD1, RAD51, MRN to regulate chromatin remodeling, exchange of information from the undamaged template, and strand resection after repair (Caestecker and Walle 2013). Both BRCA1 and BRCA2 are also critical to the protection of replication forks during the S phase (D'Andrea 2018). Cells lacking BRCA1/2 show reduced proliferation, increased chromosomal aberrations, and increased susceptibility to cancer development (Venkitaraman 2014). Chromosome instability due to HR repair pathway defects (caused by BRCA1/2 mutations) normally activates apoptosis to prevent tumorigenesis (Lee et al. 2014). However, p53 mutations and selective pressure favor uncontrolled cell proliferation, resulting in the formation of tumors despite checkpoint controls to account for DNA damage (Lee et al. 2014). In these cancer cells, HR repair dysfunction is compensated for by the activation of single-stranded DNA break repair pathways, chief among which is the BER pathway (Farmer et al. 2005). PARP activation, a result of DNA damage, is a major driver in BER (Livraghi and Garber 2015).

**Fig. 1:**
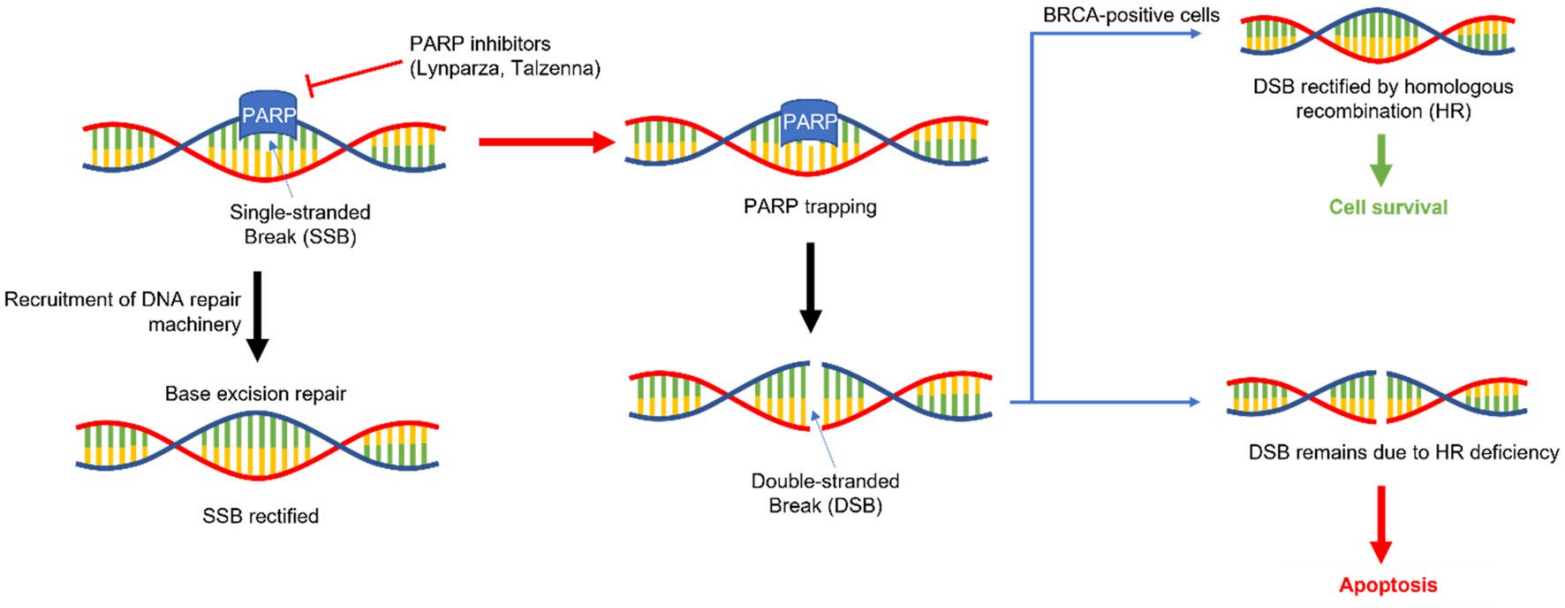
Mechanism of action of PARP inhibitors. PARP recognizes and binds to single-stranded breaks in DNA and initiates the recruitment of base excision repair (BER) machinery to repair the break. When inhibited, PARP becomes trapped at the site of the SSB, causing a double stranded break. In the absence of BRCA1/2 and homologous repair mechanisms, this break remains resulting in apoptosis downstream

PARP inhibitors like Lynparza and Talzenna function through two mechanisms (Fig. 1) (D'Andrea 2018). Firstly, the inhibition of PARP catalytic activity prevents the recruitment of DNA damage repair machinery, blocking the base excision repair process, leading to replication fork stalling during the S phase and the conversion of the single-strand nick into a double-stranded break (Fig. 1) (D'Andrea 2018). The double-stranded break is recognized by HR pathway proteins but due to defects in the pathway caused by BRCA1/2 mutations, the repair is unsuccessful, leading to apoptosis (Fig. 1) (D'Andrea 2018). A second proposed mechanism involves the prevention of PARP detachment from the DNA damage site (single and/or double-stranded) (Fig. 1) (Shen et al. 2015). The trapping of PARP at the site requires the recruitment of HR pathway machinery, which is defective in BRCA1/2-mutant breast cancer tumors (Fig. 1) (D'Andrea 2018). Interestingly, studies have indicated that Talzenna is a more effective “trapping agent” than Lynparza though both molecules have similar PARP inhibition activity (Shen et al. 2015). There are additional mechanisms that govern PARP inhibitor sensitivity in human breast tumors deficient in BRCA1/2 such as the PARP/POLQ pathway (D'Andrea 2018). However, these mechanisms are still under investigation (D'Andrea 2018).

#### Clinical application

##### Lynparza (olaparib)

In January 2018, Lynparza became the first treatment approved by the FDA for HER2-negative metastatic breast cancer patients with BRCA1/2 mutations (U.S. 2018a; Caulfield et al. 2019). To be eligible, patients are required to have undergone chemotherapy and/or hormone therapy if their tumor is hormone-positive (Caulfield et al. 2019). The National Comprehensive Cancer Network (NCCN) guidelines made Lynparza a Category 1 recommendation (Caulfield et al. 2019). The drug, manufactured by AstraZeneca, is available in tablet form and is taken in 300 mg doses twice daily until disease remission (Zimmer et al. 2018). In the phase 3 clinical trial OlympiAD, 97% of patients experienced side effects such as anemia, nausea, diarrhea, and fatigue amongst others (Robson et al. 2017). However, discontinuation due to these effects only occurred in 5% of the cohort so, overall, the drug was well tolerated (Robson et al. 2017). Lynparza’s effectiveness as a monotherapy in BRCA1/2-deficient primary TNBC tumors was demonstrated by a recent clinical trial conducted by Eikesdal et al. (2021). Patients, who had not received prior chemotherapy, with primary TNBC received Lynparza for 10 weeks (Eikesdal et al. 2021). Out of 32 patients, 18 responded to the treatment (Eikesdal et al. 2021). While Lynparza is primarily being used as a monotherapy today, clinical trials are being conducted to test its efficacy in combination with other established therapies (Zimmer et al. 2018). For example, the phase I/II trial by Dent and colleagues is currently testing the use of Lynparza with Paclitaxel in patients with metastatic triple negative breast cancer (Dent et al. 2013).

##### Talzenna (talazoparib)

In October 2018, the FDA approved an alternative PARP inhibitor for the treatment of HER2-negative metastatic breast cancers with germline BRCA mutations (U.S. 2018b). Developed and manufactured by Pfizer, clinical studies showed that Talzenna performed similarly to Lynparza (McCann 2019). Patients with BRCA-mutated triple negative breast cancer as well as those with hormone receptor-positive, HER2-negative breast cancer are eligible for treatment (McCann 2019). Talzenna is administered orally in 1-mg doses, taken once daily until disease progression is halted (McCann 2019). Its most adverse side effects include anemia, thrombocytopenia, and fatigue (Litton et al. 2018). While 98.6% of patients experienced adverse side effects, only 5.9% of this subset of patients in the EMBRACA trial discontinued treatment as a result (Litton et al. 2018). Like Lynparza, Talzenna is currently being tested in combination with cytotoxic chemotherapy in clinical trials, though results have yet to be reported (McCann 2019).

### Anthracycline-based chemotherapy

Anthracyclines are a class of drugs that act as DNA intercalating agents, thereby interfering with the activity of Topoisomerase II (Top2) in eukaryotic cells (Marinello et al. 2018). Daunomycin, the predecessor to doxorubicin, was isolated from *S. peucetius* bacteria and thereby dubbed the “antitumor antibiotic” (Arcamone et al. 1969). Doxorubicin was subsequently isolated from *S. peucetius caesius*, a variant strain developed through mutagenesis of *S. peucetius* (Arcamone et al. 1969). At the time, the development of doxorubicin allowed for the administration of lower chemotherapy doses due to its higher potency compared to daunomycin, despite the fewer side effects caused by the latter (Bonadonna et al. 1969). Shortly thereafter, in 1974, the drug was approved by the FDA for the treatment of metastatic breast cancer (Cortazar et al. 2012). In the following decades since its approval, several clinical trials, using triple negative breast cancer patients amongst others, have shown the efficacy of doxorubicin in increasing survival by 3–6 months when compared to the regimen without the drug (A'Hern et al. 1993; Paridaens et al. 2000). More recent trials are testing the efficiency of doxorubicin in combination with taxanes such as paclitaxel as well as cyclophosphamide (Bergin and Loi 2019). While the drug is widely prescribed for various cancers today, it is severely cardiotoxic in cumulative doses and is therefore administered periodically (Findlay et al. 2007). Currently, doxorubicin is manufactured by Bedford Laboratories under the brand name Adriamycin (Khasraw et al. 2012).

Epirubicin, manufactured by Pfizer under the name Ellence, was approved for the adjuvant treatment of breast cancer in 1999 (Khasraw et al. 2012). It is an epimer of doxorubicin and therefore has a similar therapeutic profile (Findlay et al. 2007). However, epirubicin has a more favorable toxicity profile due to reduced cardiac and hematologic toxicity (Khasraw et al. 2012). Therefore, epirubicin can be administered at higher doses before causing adverse cardiovascular events which may result in improved response rates (Findlay et al. 2007; Khasraw et al. 2012). A clinical trial in 2010 showed that when epirubicin was added to an adjuvant chemotherapy regimen, it resulted in a 90% recurrence-free survival rate (RFS), higher than a doxorubicin-based regimen (Burnell et al. 2010).

Overall, decades of clinical evidence and patient data have led to both doxorubicin and epirubicin becoming a major component of both early and advanced breast cancers today (Collignon et al. 2016). As epimers, they share a similar mechanism of action in their interference in topoisomerase 2 functioning and intercalation of DNA (Beretta et al. 2008).

#### Mechanism of action of anthracyclines

**Fig. 2:**
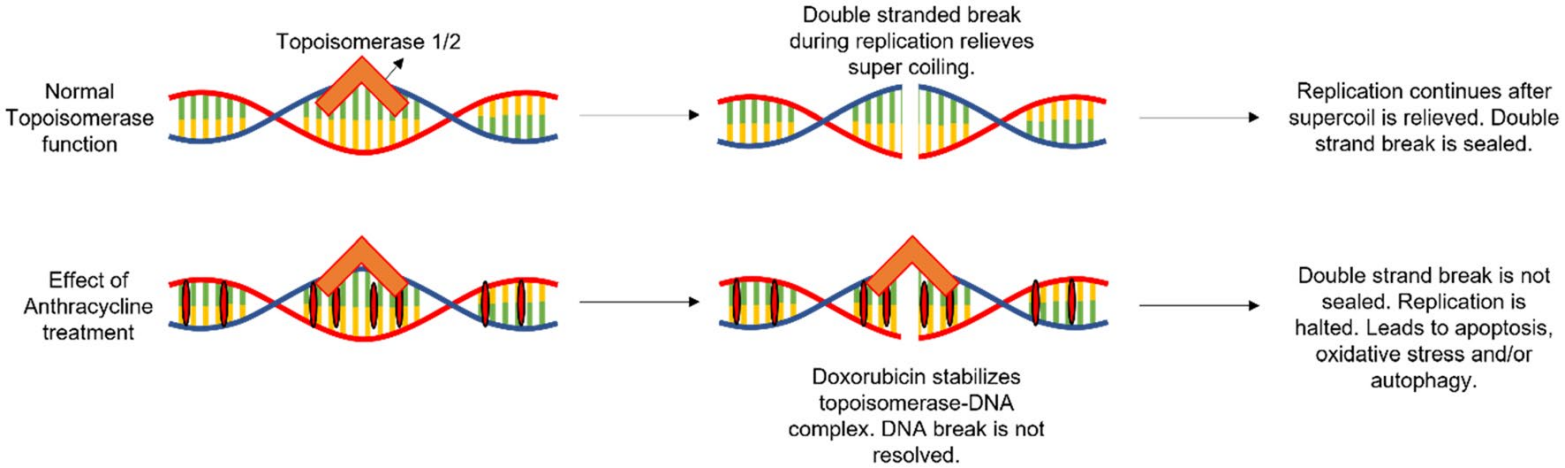
The mechanism of action of anthracyclines. Transcription and replication, the vital relieving of stress due to DNA super coiling, is conducted through the introduction of double stranded breaks by topoisomerase 1/2. These breaks are then sealed by DNA repair machinery. Doxorubicin inserts itself between DNA base pairs, thereby trapping topoisomerase 1/2 in place after it has catalyzed the double stranded break. This halts replication, leading to cell death

Anthracyclines primarily target DNA by inserting between base pairs and remaining intercalated through ionic and steric bonding (Fig. 2) (Marinello et al. 2018). However, the ability of these drugs to bind to DNA is not central to their antitumor activity (Beretta et al. 2008). Evidence suggests that these intercalating agents are cytotoxic as a result of their interference with topoisomerase II (Top2), an enzyme that regulates the supercoiling and unwinding of DNA during transcription, replication, and recombination (Fig. 2) (Nitiss 2009). Both doxorubicin and epirubicin are well documented as Top2 “poisons (Marinello et al. 2018).

Top2 enzymes create double-strand breaks in DNA, causing DNA relaxation and coiling where necessary (Fig. 2) (Nitiss 2009). They act as a homodimer of two isozymes (Top2α and Top2β) and use ATP for catalytic activity (Marinello et al. 2018). The isozymes do not differ in their activity but are differentially expressed, with Top2α highly expressed in proliferating cells and Top2β expressed at equal levels in all cells (Beretta et al. 2008). Top2β knockout in mice was found to cause prenatal death as a result of severe developmental defects affecting neuronal cells (Lyu et al. 2006). Top2α was shown to interact with and regulate the transcription of ribosomal RNA genes in highly proliferating cells, a crucial process in cell growth (Ray et al. 2013). These studies provide a context for the importance of Top2 and why interference of its activity is crucial to the cytotoxic and therapeutic effects of DNA intercalating agents such as anthracyclines.

Top2 begins its DNA cleaving activity by binding specific sites (called the G-segment), such as promoter regions or other sequences along actively transcribed genes (Marinello et al. 2018). The G-segment is nicked on each strand, resulting in a double-strand break with a 5’ overhang on the cleaved strands (Marinello et al. 2018). The active tyrosine site in the enzyme (Y782) then binds to and stabilizes the DNA termini at the break site (Beretta et al. 2008). As ATP binds to Top2, it undergoes a conformational change and passages a second, unbroken strand (T-segment) through the double-stranded break site (Beretta et al. 2008). The passage step is vital to the uncoiling of DNA as it allows for the separation of two coiled segments (Marinello et al. 2018). Finally, Top2 hydrolyzes ATP, seals the G-segment break, and resets the system, allowing for the process to repeat at a different site (Beretta et al. 2008).

Intercalating agents such as doxorubicin and epirubicin insert themselves between adjacent base pairs in a DNA sequence (Fig. 2) (Marinello et al. 2018). The drug-DNA complex effectively “traps” Top2 when the enzyme binds to the sequence and attempts to perform its function (Fig. 2) (Pommier et al. 2016). Specifically, while the planar moieties of doxorubicin and epirubicin form stacking interactions with DNA base pairs, their side chains recognize and bind to Top2 (Marinello et al. 2018). Once trapped at the binding site, the Top2-drug-DNA complex acts as an impediment to replication and transcription (Pommier et al. 2016). For example, as the replication fork approaches the Top2-drug-DNA complex, the enzymes involved in replication collide with the immovable complex, resulting in incomplete replication products (termed replication run-off) (Pommier et al. 2016). Top2 poisons such as doxorubicin and epirubicin are, therefore, particularly potent during the S phase of the cell cycle, when DNA replication and transcription are highly active (Marinello et al. 2018). The DNA fragments are, effectively, permanent double-strand breaks in DNA and are recognized as such by the DNA damage repair machinery (Pommier et al. 2016).

The DNA damage response (DDR) pathway, mediated primarily by ataxia–telangiectasia mutated (ATM) and ataxia–telangiectasia and Rad3 related (ATR) kinases, involves the activation of p53, ERK and checkpoint kinase 2 (CHEK2) (Kumari et al. 2017; Yang et al. 2016). The pathway arrests the cell cycle and activates apoptosis if DNA repair cannot occur, as is the case in most tumors with mutations in the repair pathway (Pommier et al. 2016). While there is extensive evidence for the involvement of the well-documented, canonical activated p53 pathway in doxorubicin-induced cell death, recent evidence has suggested that pERK can cause apoptosis in breast cancer cells treated with doxorubicin, regardless of the expression or functioning of p53 (Kumari et al. 2017). Direct interaction of Bim with Bcl-xl is capable of inducing cell death in prostate cancer cells treated with doxorubicin (Yang et al. 2016). Overall, the treatment of breast cancers with doxorubicin and epirubicin results in permanent DNA damage which in turn activates p53-mediated and p53-independent apoptosis pathways, resulting in cytotoxicity (Pommier et al. 2016).

#### Clinical application

##### Adriamycin (doxorubicin)

Current clinical practice in the United States, as described by the National Comprehensive Cancer Network guidelines, recommends the use of doxorubicin and liposomal doxorubicin as single agents in the treatment of triple negative tumors and tumors with germline *BRCA1/2* mutations (Gradishar et al. 2020). Doxorubicin, in a single chemotherapy agent system, is administered in 60–75 mg/m^2^ doses every 3 weeks in combination with the recommended cardio-protective drugs to account for the high risk of cardiac toxicity (26%) (Gradishar et al. 2020). Liposomal doxorubicin, a variant with a different delivery system, has a less frequent schedule (50 mg/m^2^ every 4 weeks) and a lower risk of cardiac toxicity (7%) (Gradishar et al. 2020; Rayner and Cutts 2014). Liposomal doxorubicin has been found to reduce the risk of other doxorubicin-associated side effects such as nausea, vomiting, alopecia, and neutropenia (Gradishar et al. 2020). Doxorubicin has also been recommended for combinatorial chemotherapy with cyclophosphamide or docetaxel (Gradishar et al. 2020). Two clinical trials have investigated the combinatorial efficacy of doxorubicin with paclitaxel/docetaxel and cyclophosphamide (Biganzoli et al. 2002; Nabholtz et al. 2003). In combination therapies, doxorubicin doses are reduced to 40–60 mg/m^2^ every 3–4 weeks with a maximum recommended cumulative dose of 450–500 mg/m^2^ (Gradishar et al. 2020; Rayner and Cutts 2014). Currently, the use of Adriamycin in combination with carboplatin and cyclophosphamide is being investigated in a neoadjuvant setting (McAndrew and DeMichele 2018).

##### Ellence (epirubicin)

Epirubicin was approved by the FDA for the treatment of breast cancer in 1999 and has since been tested for its efficacy both as a monotherapy and a combination therapy with taxanes (Rayner and Cutts 2014). While it shares the same mechanism of action and clinical efficacy as doxorubicin, several clinical trials comparing equimolar doses of the two drugs have found that patients treated with epirubicin reported less cardiac toxicity, nausea, alopecia, and myelosuppression (Khasraw et al. 2012). When used as a monotherapy, epirubicin can be used in higher doses than doxorubicin (Rayner and Cutts 2014). The drug is administered in 100–120 mg/m^2^ every 3–4 weeks with a cumulative dose limit of 900 mg/m^2^ (Rayner and Cutts 2014). Like doxorubicin, there are clinical trials currently testing the efficacy of epirubicin with carboplatin (McAndrew and DeMichele 2018). Additionally, a recent study is testing the combination of epirubicin and doxorubicin with the newly approved immunotherapy agent atezolizumab (U.S. 2018c).

### Immunotherapy (atezolizumab and pembrolizumab)

The lack of ER, HER2, and PR expression in triple negative breast tumors presents a significant challenge in designing targeted therapies (Katz and Alsharedi 2017). To address this, several studies have looked into specific processes that can be targeted in TNBC without a wider systemic effect (Katz and Alsharedi 2017). Gene expression and clinical data analyses of vital signaling processes in triple-negative breast cancer revealed that higher immune response levels were associated with a significantly better clinical outcome (Desmedt et al. 2008; Lehmann et al. 2011). The infiltration of CD8^+^ lymphocytes was reported to predict increased patient survival specifically in TNBC versus other subtypes of breast cancer (Liu et al. 2012). Additionally, the treatment of triple negative breast tumors with anthracycline chemotherapy has been shown to induce the immune system by activating CD8^+^ T cells which kill cancer cells (Stagg and Allard 2013). Immune response modulation, therefore, is a promising, targeted approach to the treatment of triple-negative breast tumors (Katz and Alsharedi 2017).

*Atezolizumab:* Atezolizumab (brand name: Tecentriq) was developed as an IgG1 monoclonal antibody targeting the protein PD-L1 (programmed cell death-ligand 1) to prevent its interaction with its receptor PD-1, resulting in the reversal of T-cell suppression (Schmid et al. 2018). The antibody was developed to specifically inhibit the PD-L1 to PD-1 interaction while still allowing for the alternative ligand PD-L2 to bind PD-1, thereby reducing autoimmune side effects (Herbst et al. 2014). In a randomized, phase 3 clinical trial, atezolizumab was tested on 451 patients in combination with nanoparticle albumin-bound (nab) paclitaxel (Schmid et al. 2018). Compared to patients who were treated with placebo and chemotherapy, patients treated with atezolizumab survived longer without disease progression with an objective response rate of 56% versus 45.9% (atezolizumab treatment group being higher) (Schmid et al. 2018). The success of the trial led to the approval of Tecentriq, by the FDA, for the treatment of triple negative breast cancer in May 2020 (U.S. 2020a).

*Pembrolizumab:* Pembrolizumab (brand name: Keytruda) was first developed and approved as a treatment for unresectable or metastatic melanoma (Raedler and Keytruda (Pembrolizumab) 2015). Unlike Atezolizumab, Pembrolizumab is a humanized IgG4κ antibody that targets the receptor PD-1 rather than its ligand (Kwok et al. 2016). Through the KEYNOTE-522 clinical trial, the effects of pembrolizumab treatment followed by chemotherapy in patients with early-stage TNBC were compared with placebo plus chemotherapy treatment (Schmid et al. 2020). It was found that a significant number of patients (64.8% versus 51.2%) benefited from the PD-1 antibody followed by adjuvant chemotherapy (Schmid et al. 2020). Following these results, the FDA approved Keytruda for the treatment of high-risk, early-stage triple-negative breast cancer in July 2021.

#### Mechanisms of action of atezolizumab and pembrolizumab

Immunotherapeutics, such as atezolizumab, primarily function by activating a patient’s immune system to recognize and kill cancer cells (Sun et al. 2018). However, cancer progression occurs through the evasion of the immune system (Chen et al. 2016). One of the major pathways of immune suppression involves the regulation of immune checkpoints, a series of ligand-receptor interactions which determine whether the T-cell response is activated or inhibited (Sun et al. 2018).

Both preclinical and clinical data have shown that CTLA-4 and PD-L1 are key proteins in the regulation of immune checkpoints in cancer cells and their upregulation was shown to negatively affect T-cell response to cancer progression (Ribas 2012). As atezolizumab targets the PD-L1-PD-1 axis, the focus of this section will be on PD-1 functioning and its effect on the immune system (Schmid et al. 2018).

PD-1 is a receptor, expressed on stimulated T-cells, which recognizes and binds its ligand PD-L1, thereby initiating an inhibitory signal (Ishida et al. 1992). It was initially characterized as an important regulator of programmed cell death in lymphoid cell lines that were induced to die (Ishida et al. 1992). The receptor is expressed on the surface of memory T-cells that have been previously induced with an antigen as well as on dendritic cells and natural killer cells (Akinleye and Rasool 2019). It recognizes and binds to its ligands PD-L1 and PD-L2, though evidence has shown that PD-L1 is the dominant ligand in the interaction (Sun et al. 2018). PD-L1 is primarily expressed on antigen-presenting cells, healthy tissue as well as tumor cells, and stroma (Sun et al. 2018). In a proinflammatory setting, multiple cell types tend to increase PD-L1 production in response to IFN-γ and IL4 through STAT1 activation (Sun et al. 2018; Akinleye and Rasool 2019). Indeed, this may explain the production of PD-L1 in tumor cells in response to cytokines in the tumor microenvironment (Akinleye and Rasool 2019).

Previous studies have demonstrated that PD-L1 binding to PD-1 inhibits T-cell proliferation and survival, among other effects (Butte et al. 2007; Keir et al. 2006). Specifically, the PD-1/PD-L1 axis, when induced, affects the production of proinflammatory cytokines IFN-γ, TNF-α, and IL-2 (Keir et al. 2006). In healthy tissue, PD-1/PD-L1 signaling serves to prevent autoimmunity, also known as peripheral T-cell tolerance (Keir et al. 2006). Indeed, PD-1 expression on T-cells has to be induced by antigen-presenting cells carrying antigen-MHC complexes which results in the inhibition of future immune activation events that are normally regulated through CD28 or IL-2 (Akinleye and Rasool 2019). In effect, PD-1/PD-L1 helps “check” the immune system from an overactive response to infection and/or inflammation through the inhibition of effector cell functioning through its control of cytokine production in T-cells, resulting in a signaling cascade that affects T-cell proliferation and survival (Akinleye and Rasool 2019).

To escape antitumor immune responses, cancer cells (including TNBC cells) take advantage of the PD-1/PD-L1 axis (Sun et al. 2018). PD-1 expression was found to be increased in tumor-infiltrating lymphocytes in patients with TNBC, implying the inhibited state of the immune response to cancer (Mittendorf et al. 2014). Furthermore, several cancer subtypes, including TNBC, have been shown to express higher levels of PD-L1 either through the upregulation of adaptive or innate immune resistance pathways (Akinleye and Rasool 2019). In the innate immune resistance pathway, PD-L1 expression is increased through the activation of PI3K/AKT in some cancer subtypes, regardless of the cytokines present in the tumor microenvironment (Akinleye and Rasool 2019). The inhibition of AKT was found to reduce the expression of PD-L1 in TNBC cells, implying that TNBC cells overexpress PD-L1 through this mechanism (Mittendorf et al. 2014). PD-L1 expression can also be induced through IFN-γ production in the tumor microenvironment (adaptive immune resistance) (Akinleye and Rasool 2019). The propagation of this inhibitory effect on T-cells results in dysfunction and exhaustion of the immune response to tumors (Akinleye and Rasool 2019).

**Fig. 3:**
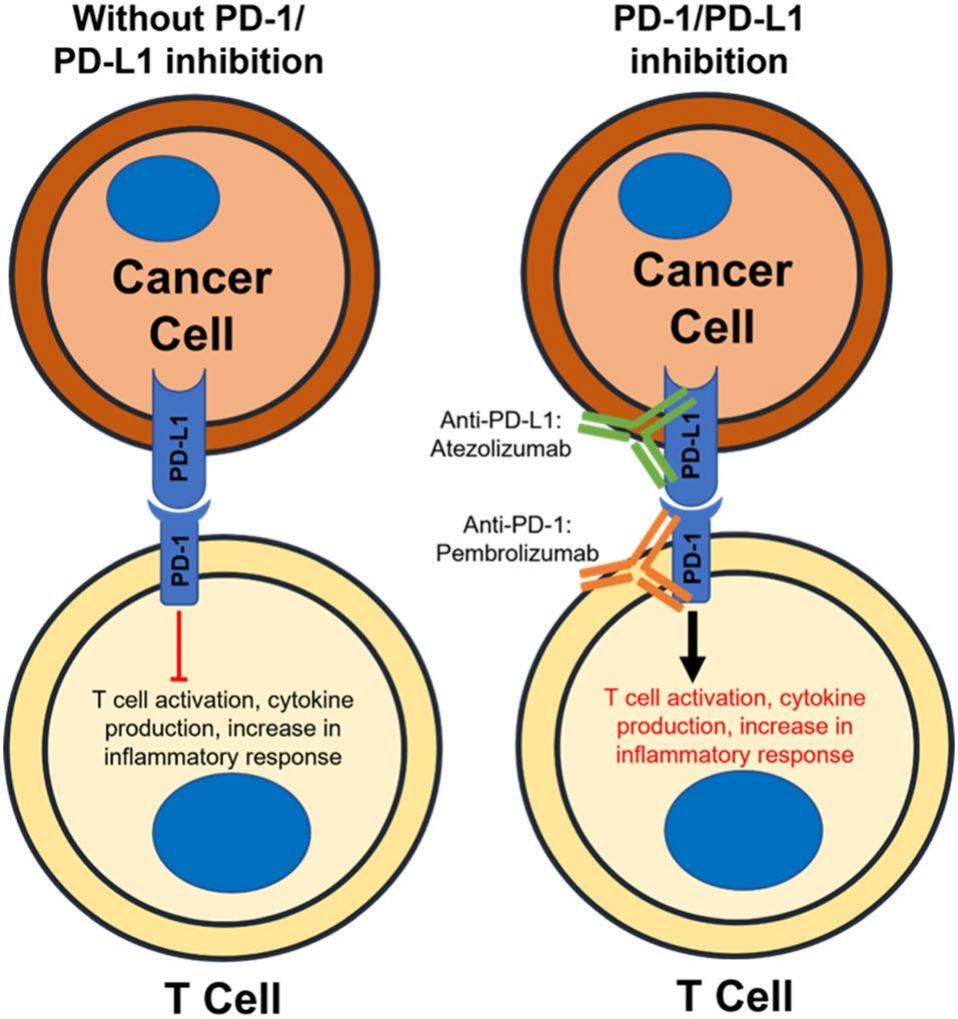
Mechanism of action of the anti-cancer activity of immunotherapeutics. The interaction of the receptor PD-1 with its ligand PD-L1 normally reduces inflammatory response. Tumour cells use this interaction to suppress anti-cancer T-cell response. Inhibition of either the receptor PD-1 (using pembrolizumab) or the ligand PD-L1 (using atezolizumab) results in the activation of the immune response against the tumour cells

As an immune checkpoint inhibitor, atezolizumab binds to PD-L1 and suppresses the functioning of the PD-1/PD-L1 axis (Fig. 3) (Schmid et al. 2018). By binding to PD-L1, atezolizumab not only prevents PD-1 activation but also allows for the B7-CD28 mediated activation of CD8^+^ T-cells in response to tumor antigens (Ribas 2012). Furthermore, PD-L1 blockade reverses CD8^+^ T-cell exhaustion, hence restoring their cancer-killing function (Pauken and Wherry 2015). Once PD-L1/PD-1 signaling is inhibited, the T-cells can respond to the inflammatory cytokines described previously to perform their anti-tumor function (Fig. 3) (Akinleye and Rasool 2019). Particularly, evidence indicates IFN-γ signaling is vital to the regulation of CD8^+^ T-cells after PD-L1 blockade (Sun et al. 2018). Further signaling changes caused by PD-L1 inhibition are currently being investigated along (Akinleye and Rasool 2019). While pembrolizumab binds to a different target, namely, the PD-1 receptor, its effect on the suppression of the PD-1/PD-L1 axis is like atezolizumab (Schmid et al. 2018). The efficacy of the two drugs in the treatment of advanced squamous non-small-cell lung cancer, in combination with chemotherapy, was compared and it was found that pembrolizumab plus chemotherapy resulted in superior overall survival and disease-free progression rates (Zhang et al. 2018). A similar comparison has not yet been made in TNBC patients.

#### Clinical application

In March 2020, Atezolizumab became the first PD-1 inhibitor to be approved by the FDA for the treatment of advanced and/or metastatic triple negative breast cancer (U.S. 2020a). The drug is now used alongside anthracycline or taxane-based chemotherapy and is administered in 840-mg doses every 2 weeks until the patient is progression-free or adverse side effects occur (Schmid et al. 2018). Patients treated with Atezolizumab and chemotherapy were shown to have increased nausea (46% versus 38%), neutropenia (20.8% versus 15.3%), and hypothyroidism (13.7% versus 3.4%) versus patients who were only treated with chemotherapy (Schmid et al. 2018). However, only 15.9% of patients in the phase 3 clinical trial withdrew from the atezolizumab treatment group versus 8.2% withdrawals in the chemotherapy-only group (Schmid et al. 2018). Currently, clinical trials are underway to test the effectiveness of combining atezolizumab with carboplatin and cyclophosphamide as well as HDAC inhibitors for the treatment of TNBC patients (U.S. 2020b, 2020c).

Pembrolizumab was approved for the treatment of high-risk, early-stage TNBC cases in July 2021 (Administration 2021). Treatments are administered at doses of 200 mg every 3 weeks or 400 mg every 6 weeks for a total of 24 weeks alongside chemotherapy (before surgery) (Schmid et al. 2020). Subsequently, Pembrolizumab treatment is continued, without accompanying chemotherapy, for 27 weeks (Schmid et al. 2020). The most common side effects observed in the clinical trial were febrile neutropenia, anemia, and pyrexia (Schmid et al. 2020).

### Taxane-based chemotherapy

In the modern clinical setting, the taxane family of chemotherapeutic drugs is one of the most effective antitumor therapies in general and particularly for triple-negative breast cancers due to the lack of expression of traditional targets (Nabholtz and Gligorov 2005). In 1971, Wani and colleagues derived paclitaxel (brand name: Taxol) from the bark extract of *Taxus brevifolia*, an evergreen yew from the Pacific Northwest, and described its antitumor and specifically, antileukemic properties (Wani, et al. 1971). Several years later, Schiff et al. discovered that Taxol reduced HeLa cell division significantly through its stabilization of microtubule assembly (Schiff et al. 1979). The limited availability of the drug led to the development and isolation of docetaxel, a semi-synthetic analog of paclitaxel from bark extracts of *Taxus baccata* (Ringel and Horwitz 1991). While structurally similar to paclitaxel barring minor chemical modifications, docetaxel was found to have a greater affinity to beta-tubulin, reduced efflux rate, and no cardiotoxic effects compared to paclitaxel (Nabholtz and Gligorov 2005).

The mechanism of action of taxanes was considered unique and therefore sparked interest in the development of paclitaxel and docetaxel for cancer therapy (Rowinsky et al. 1990). Several phase I/II clinical trials were then conducted to determine the dosage, toxicity, and efficacy of paclitaxel in treating doxorubicin/mitoxantrone-resistant metastatic breast tumors (Holmes et al. 1991; Nabholtz et al. 1996; Wilson et al. 1994; Seidman et al. 1998). Based on these and other clinical trials, the US FDA approved paclitaxel for the treatment of metastatic breast cancers which progressed despite prior anthracycline treatment (Cortazar et al. 2012). A later phase III clinical trial showed the increased effectiveness of paclitaxel in treating hormone-receptor negative (triple negative) metastatic breast cancers as well as anthracycline-resistant tumors (Henderson et al. 2003). Between 1992 and 1993, several phase I/II studies of docetaxel as a first and second-line therapy against metastatic breast cancer showed increased tumor response to the drug, especially in cases of anthracycline resistance (Nabholtz and Gligorov 2005; Oosterom 1995; Trudeau 1995). Based on a response rate of 37.9% in anthracycline-refractory tumors, the FDA granted accelerated approval for docetaxel in the treatment of metastatic breast cancer in 1996 (Cortazar et al. 2012).

The poor solubility, retention, and side effects associated with paclitaxel led to the development of a better delivery system and formulation for the drug in the form of albumin-bound paclitaxel (brand name: Abraxane) (Ibrahim et al. 2005). The formulation significantly reduced hypersensitivity, neuropathy, erythrocyte aggregation, and severe anaphylaxis while increasing drug transport efficacy by delivering paclitaxel in an albumin suspension (Ibrahim et al. 2005). A multicenter phase II clinical trial showed a 48% response rate in patients with metastatic breast cancer, no severe hypersensitivity reactions, and significant antitumor activity when used as a first-line treatment (Ibrahim et al. 2005).

#### Mechanism of action of taxanes

**Fig. 4:**
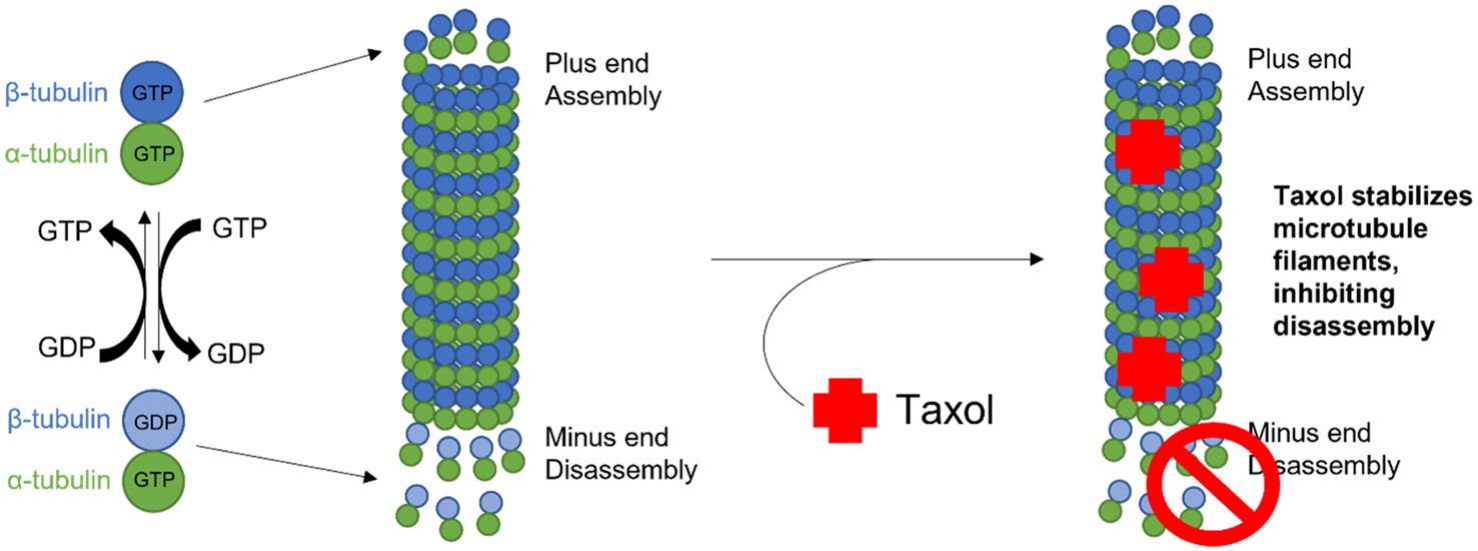
Schematic of the mechanism of action of Taxanes. By binding to microtubules, taxanes prevent their disassembly, thereby disrupting the dynamic instability of microtubules, leading to centrosomal impairment and suppression of spindle dynamics during mitosis

Unlike anthracyclines, PARP inhibitors, and immunotherapies, taxanes function through their binding to and stabilization of microtubules, preventing their disassembly into beta-tubulin components (Fig. 4) (Gallego-Jara et al. 2020). In fast-dividing cancer cells, the prevention of disassembly significantly reduces the microtubule dynamics required for cell–cell division, signaling, and migration, amongst other cellular processes (Dumontet and Jordan 2010). Therefore, there are several proposed mechanisms of action of taxol in its mediation of cell death (Gallego-Jara, et al. 2020).

Microtubules are a vital component of the cellular cytoskeleton, acting as filaments driving key processes such as intracellular transport, cell division, and polarity (Brouhard and Rice 2018). Each microtubule consists of 13 protofilaments arranged in parallel, assembled around a hollow cylindrical core (Fig. 4) (Lasser et al. 2018). These filaments are highly dynamic polymers of αβ-tubulin monomeric units and are regulated by the GTPase activity of tubulin (Fig. 4) (Akhmanova and Steinmetz 2015). While GTP binds both α and β-tubulin, primarily, GTP-bound β-tubulin is hydrolyzed during the polymerization of microtubules (Fig. 4) (Akhmanova and Steinmetz 2015). GDP-bound β-tubulin has reduced affinity to surrounding tubulin units, favoring depolymerization and leading to the dynamic behavior of microtubules where GDP-tubulin is constantly lost at one end while being replaced by GTP-tubulin at the other end (Fig. 4) (Akhmanova and Steinmetz 2015). If the rate of GTP-tubulin addition exceeds the rate of GDP-tubulin dissociation, the microtubules obtain a GTP cap (plus end) and a slow-growing minus end, thereby gaining polarity, a vital aspect of mitotic spindle formation during the cell cycle (Akhmanova and Steinmetz 2015). This dynamic instability of microtubules is vital to the cytoskeletal remodeling that occurs during mitosis as well as intracellular transportation (Brouhard and Rice 2018; Mitchison and Kirschner 1984). In rapidly dividing tumor cells, the microtubules that constitute the mitotic spindles are highly sensitive to therapeutic disruption due to their importance in sister chromatid separation (Brouhard and Rice 2018).

Taxol performs its function by stabilizing the microtubule through its binding to β-tubulin (Fig. 4) (Kellogg et al. 2017; Nogales 2000). In particular, the Taxol molecule binds to the M-loop in β-tubulin and stabilizes its association with adjacent β-tubulin molecules, thereby strengthening protofilament-protofilament interaction and reducing the rate of depolymerization caused by calcium and cold temperatures (Fig. 4) (Nogales 2000; Weaver 2014). Functionally, this causes cell cycle arrest in the G2/M-phase due to the inhibition of chromatin separation which results in the mitotic checkpoint activation and cell death thereafter (Weaver 2014; Ganguly et al. 2010; Milas et al. 1995). Induction of apoptosis through cell cycle arrest is widely regarded as the primary mechanism of action of taxanes. Paclitaxel has also been found to induce apoptosis by mediating an increase in Reactive Oxidative Species (ROS), downregulation of Bcl-2, and inhibition of the AKT/MAPK pathway to reduce cell proliferation in ovarian, canine mammary, and osteosarcoma cell lines (Strobel et al. 1996; Ren et al. 2018; Li et al. 2020). Heightened ROS levels were also found to coincide with increased expression of endoplasmic reticulum-stress proteins such as GRP78 and IRE1α in osteosarcoma cells (Li et al. 2020). The induction of endoplasmic reticulum stress then releases free Ca^2+^ and increases ROS production from mitochondria damaged by the calcium ion overload (Csordás and Hajnóczky 2009). Together, these effects initiate cytochrome C release as well as caspase 3 cleavage, both of which are mechanisms of mitochondria-mediated apoptosis (Suh et al. 2013). Additional research has shown that the induction of autophagy may be an alternative mechanism for taxol functioning. In gastric cancer cells, paclitaxel treatment demonstrated the inhibition of proliferation and induction of autophagy through p62 protein degradation (Yu et al. 2017). In non-small-cell lung cancer cells, the promotion of autophagy through esomeprazole treatment reversed taxol resistance, specifically through the reduction of intracellular pH and inhibition V-ATPase (Bai et al. 2021). However, these results are contradicted by others who have shown that autophagy inhibition reverses taxol resistance and induces caspase-dependent apoptosis (Peng et al. 2014; Kim et al. 2013; Zamora et al. 2019; Song et al. 2017).

#### Clinical application

##### Paclitaxel (taxol)

Paclitaxel is currently used to treat HR-negative, HER2-negative breast tumors as well as tumors with BRCA 1/2 germline mutations (Gradishar et al. 2020). Current guidelines recommend the use of single chemotherapy agents to mitigate the adverse side effects that affect patients. Paclitaxel, in particular, is effective in either weekly doses at 80 mg/m^2^ or every 3 weeks at 175 mg/m^2^ (Gradishar et al. 2020). Clinical trials have shown that the weekly regimen appears to improve overall survival while preserving the same response rate as the 3-weekly approach (Mauri et al. 2010).

Nab-paclitaxel, an alternative form of paclitaxel, consists of albumin-bound paclitaxel nanoparticles that have a mean diameter of 130 nm (Schettini et al. 2016). It was developed primarily to reduce the adverse side effects otherwise caused by the paclitaxel solvent (Gallego-Jara et al. 2020). The conjugation of paclitaxel to albumin allows for the rapid delivery of the drug through the gp60/caveolin-1 receptor pathway in tumor cells, resulting in greater drug penetration as well as higher maximum tolerable doses in patients (Schettini et al. 2016). Indeed, phase 3 clinical trials have shown that weekly nab-paclitaxel doses of 125 mg/m^2^ have improved patient survival rates and reduced adverse side effects such as hypertension and neutropenia (Untch et al. 2016; Gradishar et al. 2005). Currently, this regimen is the recommended scheme for the treatment of triple-negative breast tumors and can be used instead of paclitaxel or docetaxel regimens (Gradishar et al. 2020).

##### Docetaxel (taxotere)

Approved by the FDA in 1996 for the treatment of metastatic and triple negative breast tumors, docetaxel has since been used either as a monotherapy or in combination with anthracyclines in the treatment of triple negative breast cancers (Rayner and Cutts 2014; Nabholtz and Gligorov 2005). Indeed, the greater efficacy of docetaxel compared to paclitaxel has made it a safer alternative for patients as it performs the same function as paclitaxel at a lower effective dose (Nabholtz and Gligorov 2005). Currently, docetaxel is administered in 60–100 mg/m^2^ doses every 3 weeks (there appears to be no discernable difference between weekly and 3-weekly treatment cycles) in both neoadjuvant and adjuvant settings (Mauri et al. 2010). An ongoing clinical trial is testing the effectiveness of combining 75 mg/m^2^ docetaxel doses with carboplatin, a platinum-based chemotherapeutic, every 3 weeks (Ademuyiwa et al. 2021).

### Antibody-drug conjugate therapy

The attachment of antibodies and drugs was developed to efficiently deliver small molecule inhibitor molecules to cancer cells specifically (Nagayama et al. 2020). The monoclonal antibody component of the complex ensures the specificity of drug delivery and therefore increases the potency of the treatment while also reducing toxicity to healthy tissue (Nagayama et al. 2020). By recognizing and binding to an antigen-specific to cancer cells, the monoclonal antibody can then drive changes in tumor cell signaling or induce an immune response against the tumor (Chau et al. 2019). Currently, there are approximately 30 FDA-approved monoclonal antibody therapies against cancer in the market (Carter and Lazar 2018). These antibodies can be conjugated with effector molecules, such as small molecule inhibitors, cytotoxins, and radioactive isotopes, using a linker region that is cleaved at the site of the tumor thereby releasing the drug (Chau et al. 2019). The drug is then absorbed by the tumor cells, where it induces cell death (Chau et al. 2019).

One of the first antibody–drug conjugates (ADCs), gemtuzumab ozagamicin, was approved by the FDA for the treatment of patients with acute myeloid leukemia in 2000 (Nagayama et al. 2020; Sievers et al. 2001; Bross et al. 2001). Unfortunately, as the therapy did not significantly improve patient survival and caused increased off-target toxicity, it was removed from the market in 2010 and a lower dose of the drug was approved for use in 2017 (Nagayama et al. 2020). This showed that while ADCs theoretically seem straightforward to develop, there are significant challenges that limit the potency and specificity of these therapies (Chau et al. 2019). Current ADCs use a variety of cytotoxic “payload” molecules such as calicheamicins or SN38 (DNA-damaging agents), maytansines, or auristatins (anti-tubulin agents) or antitumor antibiotics (Nagayama et al. 2020; Chau et al. 2019). The next generation of ADCs included brentuximab vedotin, used to treat Hodgkin’s lymphoma and anaplastic large-cell lymphoma, and trastuzumab emtansine, used for HER2-positive breast cancer treatment (Nagayama et al. 2020). Unlike first-generation ADCs, these therapies were effective in reducing toxicity while also improving overall patient survival (Nagayama et al. 2020).

The development of ADCs for the treatment of patients with TNBC has been summarized by Nagayama and colleagues (Nagayama et al. 2020). Of the ADCs that have been tested, sacituzumab govitecan-hziy (brand name: Trodelvy) was approved by the FDA, in 2021, for the treatment of metastatic TNBC patients who had received at least two treatments previously (Bardia et al. 2021). In a phase 3 clinical trial, Sacituzumab govitecan treatment was compared to chemotherapy in patients with metastatic, treatment-refractory, TNBC (Bardia et al. 2021). Overall survival among patients treated with the ADC was 12.1 months versus 6.7 months for those treated with chemotherapy, displaying an objective response rate of 35% versus 5% respectively (Bardia et al. 2021). These significant improvements made by the administration of Trodelvy led to its approval by the FDA.

#### Mechanism of action of sacituzumab govitecan

**Fig. 5:**
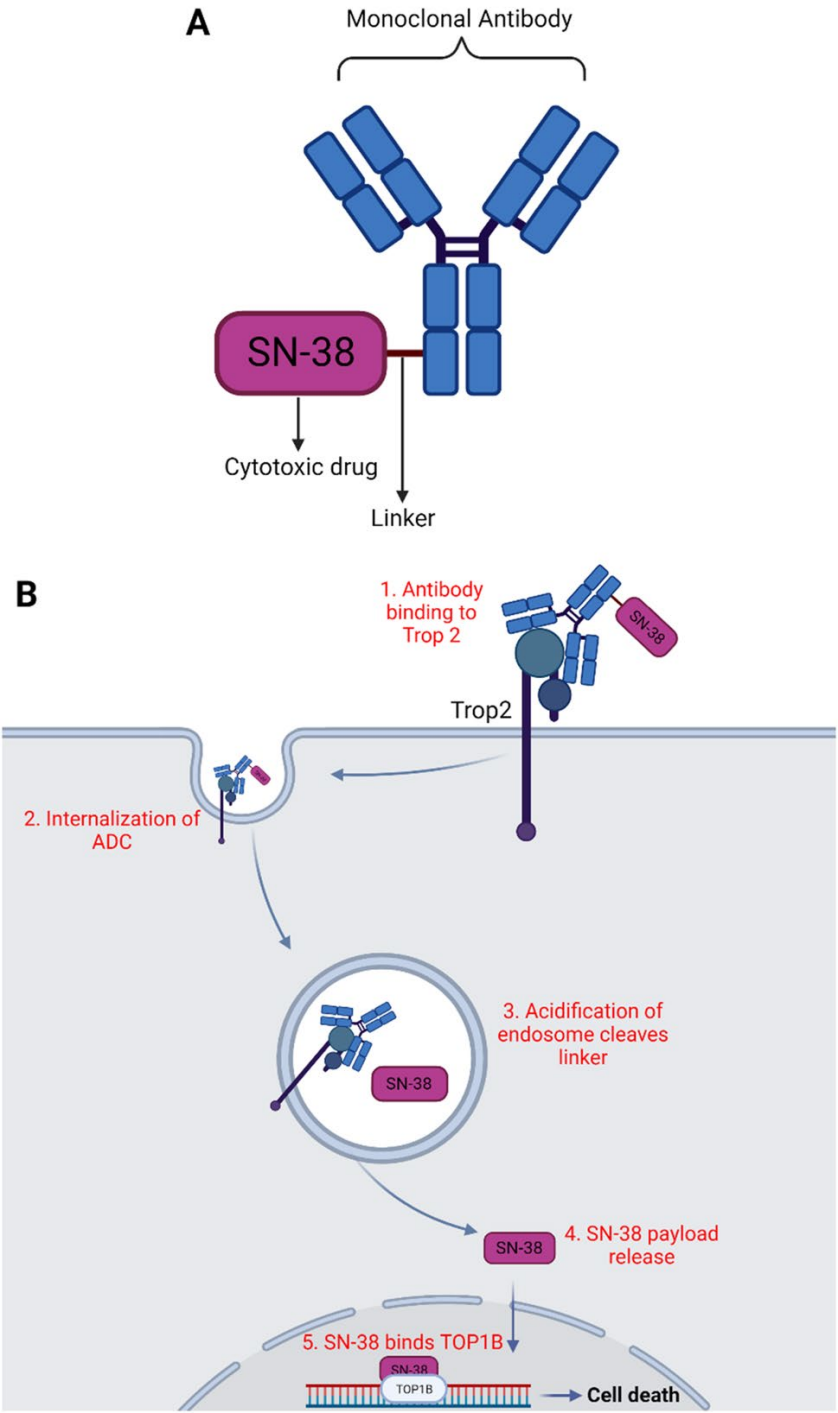
Schematic of sacituzumab govitecan and its mechanism of action. **a** Representation of the 3 main components of sacituzumab govitecan, the antibody, the cytotoxic payload and the linker between them. **b** The mechanism of action of Trodelvy in a triple negative breast cancer cell. The antibody recognizes and binds to Trop2, is then internalized into the cell. This process induces the cleavage of the linker, releasing SN-38 into the cytoplasm after which it binds to and inhibits TOP1B, causing double stranded DNA breaks and cell death thereafter. Created with Biorender.com

In general antibody–drug conjugates consist of 3 components: an antibody against a target specific to cancer cells, an anti-cancer cytotoxic drug, and a linker region to conjugate the two (Fig. 5a) (Chau et al. 2019). Sacituzumab govitecan is an ADC consisting of an anti-Trop2 monoclonal antibody conjugated with the cytotoxic drug SN-38 (a topoisomerase I inhibitor) through a proprietary, pH-sensitive, cleavable linker (Fig. 5a) (Bardia et al. 2021; Moon et al. 2008). While the therapy was designed to target tumors expressing high levels of Trop2, the Phase III clinical trial by Bardia and colleagues showed that sacituzumab govitecan was significantly beneficial to patients with metastatic TNBC, regardless of Trop2 expression, though a greater benefit was found in patients with high Trop2-expression tumors (Bardia et al. 2021). In tumors expressing Trop2, the antibody component of sacituzumab govitecan would be able to recognize the surface protein and bind to it, resulting in the internalization of the ADC through the formation of an endosome (Fig. 5b) (Bravaccini and Maltoni 2021). The subsequent acidification of the endosome results in the cleavage of the linker, thereby releasing the drug, SN-38, into the cytoplasm upon the fusion of the endosome with the lysosome (Fig. 5b) (Nagayama et al. 2020). As a topoisomerase I inhibitor, SN-38 prevents the repair of single-strand breaks in DNA, resulting in DNA damage and cell death thereafter (Fig. 5b) (Bravaccini and Maltoni 2021). Therefore, the mechanism of action of an ADC is dependent on the selection of an antigen that is highly expressed in cancer tissue while having reduced expression in the surrounding normal tissue (Nagayama et al. 2020). Sacituzumab-govitecan targets the antigen Trop2, a glycoprotein that is highly expressed in TNBC tissue (Bardia et al. 2021).

Tumor-associated calcium signal transducer 2 (Trop2) is a transmembrane glycoprotein that plays a role in calcium signaling and interacts with signaling regulators such as insulin-like growth factor 1 (IGF1), protein kinase C, and cyclin D1 (Goldenberg et al. 2018). It is primarily associated with cell migration, proliferation, and anchorage-independent growth in cancer cells (Nagayama et al. 2020). Since its discovery 40 years ago, it has been known by multiple names including trophoblast cell-surface antigen 2, membrane component chromosome 1 surface marker 1, gastrointestinal antigen 733–1, and epithelial glycoprotein-1 (Goldenberg et al. 2018). In a study conducted in mice, the Goldenberg group identified mouse monoclonal antibodies that bound to Trop2 in cancerous lung, breast, colon, kidney, and ovarian tissues, thereby indicating that the glycoprotein was widely expressed by multiple cancer types (Stein et al. 1990). In patients with these cancers, including TNBC, increased Trop2 expression correlates with worse prognoses (Stepan et al. 2011). Notably, Trop2 overexpression has been observed in over 80% of TNBC cases while surrounding non-cancerous breast tissue expresses lower levels of the glycoprotein (Son et al. 2018). Thus, the differential expression of Trop2 could be used to target cancer cells with antibodies and deliver drugs specifically while reducing off-target effects (Goldenberg et al. 2018). To this end, the humanized monoclonal antibody (known as hRS7) against Trop2 was developed to specifically recognize and bind to TNBC cancer cells that expressed Trop2 (Bardia et al. 2021).

The payload of Trodelvy is SN-38, the active ingredient in irinotecan, which itself is a well-known inhibitor of topoisomerase I and causes DNA damage (Goldenberg and Sharkey 2019). While irinotecan is highly potent against various human cancer cell lines, with its IC_50_ in the nanomolar range, its low bioavailability presented challenges to its therapeutic application (Goldenberg and Sharkey 2019; Sharkey et al. 2015). Particularly, the conversion of irinotecan to its active form SN-38 within a patient’s liver, intestine or plasma was highly inefficient (Sharkey et al. 2015). Therefore, the active metabolite SN-38 was directly conjugated to the antibody at a ratio of 7.6 molecules of SN-38 for every 1 molecule of the hRS7 antibody (Goldenberg and Sharkey 2019). Upon internalization and release into the cell, SN38 binds to and stabilizes topoisomerase IB, forming an SN-38-TOPIB-DNA complex (Peters and Chapter 2020). This prevents TOPIB-induced single-stranded DNA breaks from repairing and when the DNA replication fork (during S-phase) encounters the complex, irreversible double-stranded breaks are formed, leading to cell death as a result (Fig. 5b) (Peters and Chapter, 2020).

It should be noted that while this mechanism of action is similar to that of anthracyclines, SN-38 targets topoisomerase I, which catalyzes single-stranded DNA breaks while anthracyclines target topoisomerase II, which catalyzes double-stranded DNA breaks. Interestingly, however, p53-mediated apoptosis appears to be a common mechanism through which both SN38 and anthracyclines mediate their cytotoxic function (Takeba et al. 2007; Derenzini et al. 2009).

#### Clinical application

Currently, Trodelvy has been indicated for the treatment of metastatic TNBC patients who have previously received at least 2 therapies, one of which for the metastasis itself (Bardia et al. 2021). A dose of 10 mg per kg is injected intravenously once a week in 21-day treatment cycles until disease progression or intolerable toxicity occurs (Bardia et al. 2021). The most common adverse side effects include neutropenia, diarrhea, nausea, fatigue, and anemia (Bardia et al. 2021).

## Conclusion and perspectives

The emergence of therapies such as Trodelvy, olaparib, and Keytruda, an antibody–drug conjugate, a PARP inhibitor, and an immunotherapeutic respectively, can be traced back to an increase in understanding, not only of the tumor microenvironment but of the signaling pathways that affect tumor growth. These drugs represent newer, more targeted tools that clinicians can now use to combat an otherwise treatment-refractory disease in TNBC in a manner that reduces patient risk while not compromising on reducing tumor recurrence. The approval of these agents by the FDA, either as mono or combinatorial therapies, allows for a more nuanced approach to the treatment of TNBC.

While chemotherapy remains the standard, not only in TNBC treatment but also in most cancers, the emergence of new drug delivery methods, as shown by nab-paclitaxel, will help reduce the adverse side effects normally associated with this class of anti-cancer agents. The emerging use of nano-delivery systems may be the key to increasing the pharmacokinetic efficiency of existing, approved therapies thereby reducing systemic toxicity and circumventing drug resistance, a problem that has plagued chemotherapy use for decades (Yao et al. 2020).
